# Molecular-based evidence for school transmission of enteroaggregative *Escherichia coli* among apparently healthy children attending nursery, infant, and primary schools in Madrid (Spain)

**DOI:** 10.1007/s00431-025-06430-z

**Published:** 2025-10-04

**Authors:** María Teresa Llorente, Aly Salimo Muadica, Alejandro Dashti, Pamela Carolina Köster, Begoña Bailo, Andrea López, David Carmena, Sergio Sánchez

**Affiliations:** 1 Special Pathogens Reference and Research Laboratory, National Centre for Microbiology, Institute of Health Carlos III, 28220 Majadahonda, Madrid Spain; 2 Parasitology Reference and Research Laboratory, National Centre for Microbiology, Institute of Health Carlos III, 28220 Majadahonda, Madrid Spain; 3 Faculty of Health Sciences, Alfonso X El Sabio University (UAX), 28691 Villanueva de la Cañada, Spain; 4 Women for Africa Foundation, 28046 Madrid, Spain; 5 National Centre for Microbiology, Institute of Health Carlos III, 28220 Majadahonda, Madrid Spain; 6 CIBER Infectious Diseases (CIBERINFEC), Institute of Health Carlos III, Madrid, Spain

**Keywords:** Enteroaggregative *Escherichia coli*, Children, School, Prevalence, Transmission, Whole-genome sequencing

## Abstract

Information on the epidemiology, transmission dynamics, and public health impact of enteroaggregative *Escherichia coli* (EAEC) infection in schoolchildren from high-income countries is scarce. This study investigated the occurrence of EAEC infections in apparently healthy children (0–12 years) attending nursery, infant, and primary schools in Spain. High-resolution whole-genome sequencing typing was used to detect and trace back unnoticed episodes of transmission within school settings. An overall EAEC prevalence of 5.1% was observed, with children in the 0–3 age group showing the highest prevalence (24.2%). Besides their gastrointestinal potential, 17% of EAEC isolates revealed an additional urinary/systemic pathogenic potential. Presumptive outbreaks of EAEC infection were identified in two different nursery schools involving the endemic subtypes O126:H27-ST200 (15 children) and O111:H21-ST40 (12 children). Most affected children shared caregivers and common areas including activity, eating, sleeping, and diapering/toileting rooms. Direct person-to-person transmission was highly suspected, although foodborne transmission could not be completely ruled out. Six independent micro-foci of EAEC infections were additionally identified in five different infant and primary schools also involving O126:H27-ST200 (two children) and O111:H21-ST40 (three children), as well as O3:H2-ST10 (three children), O44:H18-ST1380 (two children and two siblings), and ONT:H33-ST34 (four children). No clear information was available on the sources of infection and transmission routes in these settings. *Conclusion*: Apparently healthy Spanish schoolchildren may be carriers and potential spreaders of certain EAEC subtypes with gastrointestinal/extra-intestinal pathogenic potential. While transmission within school settings appears to be the most likely explanation for the EAEC genomic clusters identified, particularly among toddlers, extra-school infections through alternative pathways cannot be entirely ruled out.

**Table:** 

**What is Known:**	
• *EAEC is increasingly considered as an important agent of domestically acquired paediatric diarrhoea in high-income countries.*	
• *Endemic EAEC subtypes differ between low- and high-income countries.*	
**What is New:**	
• *Apparently healthy children in high-income countries may be carriers and potential spreaders of certain EAEC subtypes with gastrointestinal/extra-intestinal pathogenic potential.*	
• *Transmission of endemic EAEC subtypes can occur within school settings, particularly during early childhood, without precluding other transmission modes.*

**Supplementary Information:**

The online version contains supplementary material available at 10.1007/s00431-025-06430-z.

## Introduction

Enteroaggregative *Escherichia coli* (EAEC) is a major contributor of paediatric diarrhoea in low-income countries [1, 2] and a leading cause of travellers’ diarrhoea [3]. However, EAEC has been increasingly associated with domestically acquired gastrointestinal illness also in high-income countries, particularly among children [4–8]. In these settings, EAEC prevalence ranges from 1.9% to 5.9% in the general population [4, 9–13] and up to 11.9% in children ≤ 5 years old [4, 5, 7]. In Spain, although EAEC infections are not notifiable and no surveillance has been conducted to date, EAEC has been demonstrated as an important domestically acquired bacterium in non-travel-related diarrhoeal episodes, with prevalences of 7.8% and 9.8% in the general population and children ≤ 5 years old, respectively [14]. Besides its role as an enteric pathogen, EAEC has emerged as a causative agent of UTI and systemic infections in the last years [14, 15].

EAEC typically attach to human epithelial cells in a characteristic “stacked-brick” pattern termed aggregative adherence [16]. Most EAEC strains harbour a transcriptional activator called AggR [17], which controls the expression of a large number and variety of plasmid-borne and chromosomal putative virulence genes. Among the AggR-dependent factors are five known variants of the AAF (designated I–V) [18–22] and *aatA* [23]. Such strains carrying AggR or its components have been termed typical EAEC [24]. Aggregative adherence has also been shown to be mediated by other adhesion factors such as CS22 [15] and AFP [25].

In the absence of a known non-human reservoir for EAEC, direct person-to-person transmission is thought to be most likely, particularly among young children [26]. Exposure to contaminated food and drinking water can also be significant transmission routes in some settings [27]. Indeed, EAEC has shown considerable potential for foodborne transmission in several outbreaks of gastroenteritis documented in high-income countries like Japan, Italy, and the UK [28–31]. In this context, EFSA has highlighted the convenience of routinely testing for EAEC when investigating foodborne outbreaks [32].

Clinical manifestations of EAEC infections include watery diarrhoea and occasionally mucoid diarrhoea, nausea, anorexia, low-grade fever, borborygmi, and tenesmus [27]. Cases of both acute and persistent diarrhoea have been described, with the latter being most frequently reported in children ≤ 1 year [27]. Besides their clinical impact, asymptomatic carriage of EAEC has also been reported, especially among young children [33, 34], a group more likely to shed the bacteria in their faeces for a prolonged period of time [33] and also a group in which maintaining optimal hygiene can be challenging, particularly in busy daycare facilities. However, there is little information on the prevalence of asymptomatic/subclinical EAEC infections in children from high-income countries and their transmission dynamics at school. Here we investigated the occurrence of EAEC infections in apparently healthy children aged from 0 to 12 years attending the first educational stages (nursery, infant, and primary schools) in Spain. To reveal possible unnoticed episodes of transmission within school settings, we molecularly characterized the resulting EAEC isolates and investigated their genetic relationships using WGS.

## Materials and methods

### Design and study areas

A cross-sectional study was conducted among apparently healthy children attending nursery (0–3 years old), infant (4–5 years old), and primary (6–12 years old) schools in the Madrid area, central Spain. Children attending four public nursery schools (size range: 82–190 schoolchildren) in Majadahonda (northwestern metropolitan area of Madrid) were sampled between February and March 2020. The median participation rate was 37% (range: 7–59%). Children attending public (*n* = 8) and private (*n* = 3) infant and primary schools (size range: 180–990 schoolchildren) in Leganés (southern metropolitan area of Madrid, central Spain) were sampled between January and June 2018. The median participation rate was 25% (range 12–40%).

### Sampling

Informative meetings were held for interested families, which were provided with sampling kits (sterile polystyrene plastic flask with spatula and a unique identification number) to obtain individual stool samples. Signed informed consents were obtained from parents/legal guardians, who assisted in collecting the stool samples from children and brought them to school. Samples were transported to the Spanish National Centre for Microbiology at scheduled times (usually 2–3 days after kits were provided in infant and primary schools, 5–7 days in nursery schools) and stored at 4 °C (1–5 days) or − 20 °C (> 5 days) without preservatives until further analyses. Only children providing samples and signed informed consents were included in the survey.

### Questionnaire survey

Standardized questionnaires ([35], Table S1) were provided as part of the sampling kit to be completed by the children’s parents/legal guardians. Questions included demographic characteristics (age, sex, number of siblings), behavioural habits such as hand and fruit/vegetable washing, and occurrence of diarrhoea episodes in the participant, family members, school mates, and/or pets. Additional questions addressed potential risk factors such as types of drinking water, swimming in pools or natural waters, contact with pets, and recent travel abroad. Completed questionnaires and signed informed consents were returned together with the stool samples as explained above. Only children providing stool samples, fully completed questionnaires, and signed informed consents were included in the survey.

### Microbiological analysis

Upon receipt, stool samples were processed for EAEC detection and isolation as described elsewhere [14]. Due to the lack of a formally recognized molecular definition for EAEC and considering its historical specificity [5, 36], our marker for EAEC infection was *aatA* gene. Therefore, an EAEC-positive case was defined as a child whose stool sample tested positive for *aatA* by PCR, regardless of whether or not the corresponding EAEC isolate was ultimately obtained by culturing the sample. Primer pairs and PCR cycling conditions of the in-house conventional PCR assay used for EAEC detection are shown in Table S2. A detailed description of the microbiological analysis is shown in the Supplementary methods.

### Molecular characterization using whole-genome sequencing

Genomic DNA was purified from the EAEC isolates using the NZY Tissue gDNA Isolation Kit (NZYTech, Lisbon, Portugal). A DNA library was generated using the Nextera XT DNA Sample Preparation Kit or Nextera DNA Flex Library Preparation Kit (Illumina, San Diego, CA, USA) and WGS was performed with the NextSeq 500 or NovaSeq 6000 platforms (Illumina) using 300 cycles and generating 150-bp paired-end reads. The reads were trimmed and filtered according to quality criteria and subsequently analysed for characterization purposes with an in-house pipeline as previously described [14]. A detailed description of the molecular characterization is shown in the Supplementary methods.

### Phylogenetic analysis of EAEC isolates

To reveal possible episodes of transmission within school settings, a SNP analysis was performed for all EAEC isolates belonging to the same serogenotype-ST combinations and from all settings with an in-house pipeline as previously described [14], including unrelated isolates of the same serogenotype-ST combination obtained in a previous study [14]. A detailed description of the phylogenetic analysis is shown in the Supplementary methods.

### Statistical analysis

Proportions were compared by a two-tailed chi-square test and odds ratios with 95% confidence intervals were determined using the website tool www.openepi.com. A *p* value < 0.05 was considered statistically significant.

## Results

### Detection of EAEC in the study populations

The study population consisted of 1463 children including 161 individuals attending nursery schools (children 0‒3 years of age; male/female ratio: 1.16) and 1302 individuals attending infant and primary schools (children 4‒12 years of age; male/female ratio: 1.20). Although this was an apparently healthy population, 13.3% (21/158) of children attending nursery schools and 4.4% (57/1297) of those attending infant and primary schools reported having diarrhoea in the seven days prior sampling (Tables S3 and S4). EAEC was detected in the stool samples of 5.1% (75/1463) of children, with prevalence being significantly higher in children aged from 0 to 3 years than in children aged from 4 to 12 years (24.2%, 39/161, vs. 2.8%, 36/1302; OR = 11.2, 95% CI: 6.9–18.4; *p* < 0.001). EAEC was detected in the four participating nursery schools (prevalence range: 2.5–50.0%) and in 10 out of the 11 participating infant and primary schools (prevalence range: 0–11.5%). EAEC could only be detected in 9 out of the 78 episodes of diarrhoea reported by the participants, with the remaining 66 EAEC-positive cases being asymptomatic (Tables S3 and S4).

### WGS-derived characterization of EAEC isolates

**Fig. 1:**
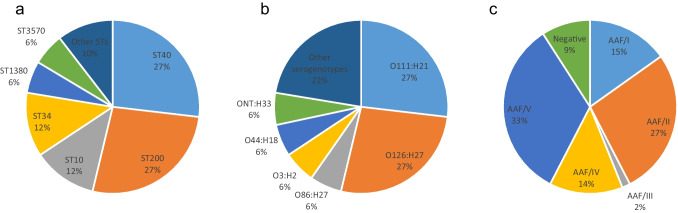
Distribution of different sequence types (ST) (**a**), serogenotypes (**b**), and aggregative adherence fimbriae (AAF) variants (**c**) among enteroaggregative *Escherichia coli* isolates. Other serogenotypes and STs (with less than three isolates each) are summarized in Tables S5 and S6. When O antigen was not predicted, it was considered non-typeable (ONT)

EAEC was isolated from 88% (66/75) of the EAEC-positive samples. The main characteristics of the 66 resulting EAEC isolates are summarized in Tables S5 and S6. Eleven distinct STs and 16 distinct serogenotypes were found. The most common STs were ST40 and ST200 (27% each), followed by ST10, and ST34 (12% each). The predominant serogenotypes were O111:H21 and O126:H27 (27% each), followed by O86:H27, O3:H2, O44:H18, and ONT:H33 (6% each) (Fig. 1, Tables S5 and S6).

Regarding the distribution of EAEC-associated putative virulence genes, all the resulting EAEC isolates harboured the AggR-regulated genes *aatA* (per study protocol) and *aap*, and therefore they can be considered typical EAEC, according to the formal definition [24]. Other frequently detected genes known to comprise the AggR regulon were *aggR* (91%), *aaiC* (79%), and *aar* (77%) (Tables S5 and S6). Evidence of a known AAF variant was found in 91% of the isolates, with AAF/V being the most frequently observed (33%), followed by AAF/II (27%), AAF/I (15%), AAF/IV (14%), and AAFIII (1%) (Fig. 1, Tables S5 and S6). Six isolates (9%) were negative for any genes attributed to the known AAF variants (also negative for CS22), but all of them showed genes encoding AFP (Table S5). Apart from EAEC-associated putative virulence genes, a substantial number of genes typically associated with ExPEC were detected among EAEC isolates (Tables S5 and S6). In particular, four EAEC isolates were additionally classified as presumptive ExPEC, three isolates were classified as UPEC, and four isolates were classified as both ExPEC and UPEC (Tables S5 and S6). Therefore, 17% of EAEC isolates in our collection revealed an additional urinary/systemic pathogenic potential.

### Detection of possible episodes of EAEC transmission within school settings

The molecular characterization of isolates (serogenotype-ST combination) together with their epidemiological relationships (the school of origin) suggested possible unnoticed episodes of transmission of different EAEC subtypes (Tables S5 and S6). Genetic relationships between epidemiologically related isolates of the same serogenotype-ST combination could be subsequently confirmed from the specific whole-genome phylogenies for each subtype. According to criteria proposed by Pightling et al. [37] for interpreting WGS analyses of foodborne bacteria for outbreak investigations, monophyletic groups of *E. coli* isolates with a median pairwise distance of 20 or fewer SNPs, a bootstrap support of 90 or higher, and some epidemiological evidence support transmission episodes.

**Fig. 2:**
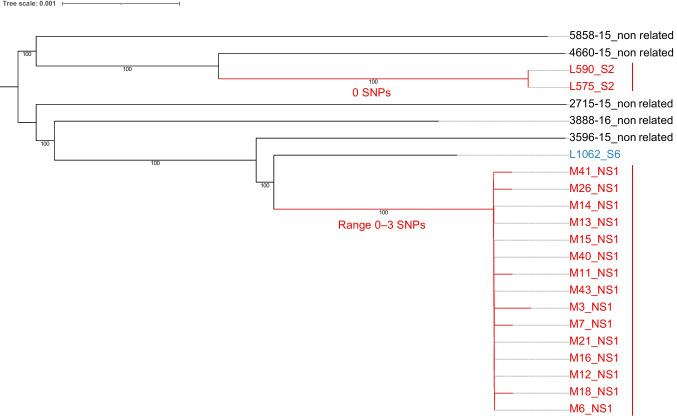
Phylogenomic analysis of the O126:H27-ST200 enteroaggregative *Escherichia coli* genomes. A SNP matrix was generated for 23 isolates with CSI Phylogeny 1.4 (https://cge.food.dtu.dk/services/CSIPhylogeny) and the published genome of *E. coli* strain A41 (accession no. NZ_CP028735.1) as a reference, according to KmerFinder 3.2 results. The SNP matrix was phylogenetically analyzed with RAxML 8.2.12 with a GTR model. Branch labels indicate support values for 1000 bootstrap replicates. Bootstrap values less than 90 are not shown. Monophyletic groups of isolates with a median pairwise distance of 20 or fewer SNPs, a bootstrap support of 90 or higher, and some epidemiological evidence supporting episodes of EAEC transmission are coloured in red. Unrelated study isolates are coloured in blue. Unrelated isolates from Llorente et al., available at https://doi.org/10.3389/fmicb.2023.1120285, are coloured in black. The tree scale indicates the distance of 0.001 nucleotide changes per site

**Fig. 3:**
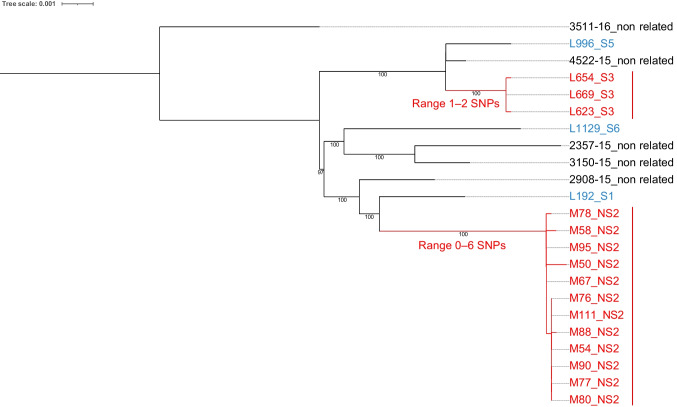
Phylogenomic analysis of the O111:H21-ST40 enteroaggregative *Escherichia coli* genomes. A SNP matrix was generated for 23 isolates with CSI Phylogeny 1.4 (https://cge.food.dtu.dk/services/CSIPhylogeny) and the published genome of *E. coli* strain ESBL 15 (accession no. NZ_CP041678.1) as a reference, according to KmerFinder 3.2 results. The SNP matrix was phylogenetically analyzed with RAxML 8.2.12 with a GTR model. Branch labels indicate support values for 1000 bootstrap replicates. Bootstrap values less than 90 are not shown. Monophyletic groups of isolates with a median pairwise distance of 20 or fewer SNPs, a bootstrap support of 90 or higher, and some epidemiological evidence supporting episodes of EAEC transmission are coloured in red. Unrelated study isolates are coloured in blue. Unrelated isolates from Llorente et al., available at https://doi.org/10.3389/fmicb.2023.1120285, are coloured in black. The tree scale indicates the distance of 0.001 nucleotide changes per site

In nursery schools (children 0‒3 years of age), WGS analyses evidenced presumptive outbreaks of EAEC infection in two different schools. In particular, EAEC O126:H27-ST200 was responsible for a presumptive outbreak within school NS1 involving 15 children from different classrooms in all educational levels (age groups 0‒1, 1‒2, and 2‒3), with 40% of them reporting diarrhoea and/or other gastrointestinal symptoms in the last 7 days (Fig. 2, Table S6). Notably, children in classrooms 1 A and 1B (ages 0–1) were cared for by the same caregivers, who were responsible for meeting their basic needs, ensuring their well-being, and supporting their developmental activities. These classrooms also shared common spaces for conducting activities, eating, sleeping, handwashing, and diapering/toileting. This was also the case for children in classrooms 1E and 1 F (age group 2‒3) (Table S6). Likewise, EAEC O111:H21-ST40 was responsible for another presumptive outbreak within school NS2 involving 12 children from different classrooms in different educational levels (age groups 1‒2 and 2‒3), with 17% of them reporting diarrhoea and/or other gastrointestinal symptoms in the last 7 days (Fig. 3, Table S6). Again, children in classrooms 2 C and 2D (age group 1‒2) shared caregivers and activities, and eating, sleeping, handwashing, and diapering/toileting areas (Table S6).

In infant and primary schools (children 4‒12 years of age), such analyses revealed six possible episodes of EAEC transmission in five different schools. In particular, EAEC O3:H2-ST10 was responsible for a transmission episode within school S1 involving three children aged 5, 8, and 10 years without any clinical manifestations (Table S5, Fig. S1). EAEC O44:H18-ST1380 was responsible for two independent transmission episodes within schools S6 and S8 involving two children aged 4 years and two siblings aged 6 and 11 years, respectively, without any clinical manifestations (Table S5, Fig. S2). EAEC ONT:H33-ST34 was responsible for a transmission episode within school S2 involving four children aged 5, 7, 8, and 9 years with one of them having reported diarrhoea in the previous 7 days (Table S5, Fig. S3). EAEC O126:H27-ST200 was responsible for a transmission episode within school S2 involving two children aged 4 years without any clinical manifestations (Fig. 2, Table S5). EAEC O111:H21-ST40 was responsible for a transmission episode within school S3 involving three children aged 4 and 5 years without any clinical manifestations (Fig. 3, Table S5).

## Discussion

This study investigated the occurrence of EAEC in apparently healthy children from 0 to 12 years of age in central Spain. An overall EAEC prevalence of 5.1% was observed, with children in the 0–3 age group showing the highest infection rate (24.2%). Strengths of the survey are (i) large sample size covering the first educational stages (nursery, infant, and primary schools) in the country, (ii) focus on apparently healthy children, a subpopulation underrepresented in previous surveys, and (iii) use of highly sensitive molecular methods including WGS to trace back transmission events within school settings.

The EAEC prevalence found here (5.1%) was in the range of those reported in previous molecular-based case–control studies conducted in paediatric populations from other high-income countries including Germany (cases: 2.0%, controls: 0.0%) [26], Switzerland (cases: 2.8–11.9%, controls: 2.2%) [5], and USA (cases: 3.1–10.0%, controls: 0.9–4.0%) [4, 6, 34, 38]. Additionally, EAEC infections have also been reported in children presenting with gastroenteritis in Spain (9.8%) [14], Italy (5.1–6.2%) [39], Israel (8.8%) [7], and USA (2.4–2.9%) [40, 41]. Of note, a 1-year longitudinal study conducted in Denmark estimated that 14% of children aged 0–6 years attending daycare facilities tested EAEC-positive at least once during the study period, although no transmission events were identified [33]. In this study, EAEC O126:H27-ST200 and O111:H21-ST40 were the most frequently identified subtypes and provided more than half of the EAEC isolates obtained. Both subtypes are among the most frequently reported in EAEC strains from Spain [14] and other high-income countries [7, 8, 33, 42–45], and even linked to outbreaks of gastroenteritis [28, 30, 46], although rarely identified among EAEC strains from low-income countries [15, 23]. Taken together, these data have remarkable public health implications, including the demonstration that EAEC infections (i) cause epidemic and non-epidemic diarrhoea in children, (ii) are endemic in high-income countries, and (iii) are relatively frequent in children without clinical manifestations who may be acting as asymptomatic carriers.

Because of EAEC infection is not regarded as a notifiable disease in Spain and it is not actively searched during outbreak investigations, identification of EAEC is typically the result of second-line detection methods or through accidental findings when looking for other causative agents [47, 48]. In this respect, our study highlights the real problem of community epidemics occurring in schools, which often remain unnoticed and unreported and for which specific recommendations in terms of control and prevention are lacking. However, we carried out an in-depth investigation to fully characterise presumptive EAEC transmission events using high-resolution WGS typing data. WGS is currently regarded by ECDC as the reference microbial typing method in outbreak investigations in the EU/EEA and increasingly applied to national surveillance of infectious diseases [49].

Our WGS analyses allowed the identification of two presumptive outbreaks of EAEC infection affecting 15 and 12 toddlers of 0–3 years of age attending two independent nursery schools. As shown in Table S6, the samples of the children involved in the presumptive outbreak by EAEC O126:H27-ST200 in school NS1 were taken over a period of 11 days. As for school NS2, the samples of the children involved in the presumptive outbreak by EAEC O111:H21-ST40 were taken over a period of 7 days. In both cases, during this sampling period, several of the children became ill with diarrhoea and/or other gastrointestinal symptoms (Table S6). Therefore, although our study was not originally intended as an outbreak investigation, we can speak of outbreaks in these cases. They were presumptive outbreaks in any case, as neither an index case nor a source of infection could be clearly established. Remarkably, 60–83% of the EAEC-positive toddlers did not report gastrointestinal manifestations in the week prior to sampling, strongly suggesting that they were asymptomatic carriers at that period. In both nursery schools there were children infected with the same EAEC strain who shared caregivers and spaces for conducting activities, eating, sleeping, handwashing, and diapering/toileting, suggesting that person-to-person transmission was the most likely mode of spread. However, foodborne transmission cannot be ruled out because (i) both nursery schools also had children infected with the same EAEC strain but not sharing caregivers or spaces and (ii) testing on caregivers, food handlers, and potentially contaminated surfaces (cooking areas, utensils) was not conduced in this survey. Unaware food handlers with poor personal hygiene may play a critical role in the unnoticed transmission of EAEC. Regardless of the case, early care and education providers should implement effective strategies to ensure proper training, adherence to hygiene standards, and regular health screenings to mitigate the risk of disease transmission to vulnerable populations like young children. Presumptive outbreaks were caused by EAEC O126:H27-ST200 and O111:H21-ST40, which have been recently identified as the most important domestically acquired EAEC subtypes in Spain and other high-income countries [14]. In particular, EAEC O111:H21-ST40, proposed to have a higher intrinsic potential to cause diarrheal disease in the UK [44], was identified as the causative agent of an outbreak of diarrhoeal illness affecting four children attending a nursery school in Belgium [47]. Other EAEC subtypes not reported here have occasionally been identified in outbreak investigations affecting children in nursery schools globally. For instance, EAEC O4 (H antigen not determined, ST not determined) was identified in 12 infants with diarrhoea in a neonatal nursery ward in Serbia [48], whereas EAEC O44:H18 (ST not determined) was found in nine children (three with diarrhoea) and four staff members in a day nursery in the UK [50].

Our WGS analyses also allowed the identification of six presumptive sporadic episodes of EAEC transmission affecting children of 4‒12 years of age attending infant and primary schools in the Leganés area. Although foodborne transmission is unlikely in these small transmission events, the fact that most of them involved children of different age groups considerably reduces the possibilities of contact between children, either in or out of school, making it difficult to establish the transmission mode and sources of infection. These micro-foci of infection involving 2–4 children were caused by EAEC O126:H27-ST200 and O111:H21-ST40 (the very same causing the presumptive outbreaks in the Majadahonda area reported above) together with O3:H2-ST10, O44:H18-ST1380, and ONT:H33-ST34. In particular, EAEC O126:NM (H antigen not determined, probably O126:H27, ST not determined) has been previously reported in four high school students with diarrhoea in Japan [30]. Other EAEC subtypes not reported here have occasionally been identified in outbreak investigations of diarrhoeal illness affecting schoolchildren globally. For instance, EAEC ONT:H10 (ST not determined) has been implicated in a massive outbreak affecting 2697 children attending elementary and junior high schools in Japan [51]. Likewise, EAEC ONT (H antigen not determined, ST not determined) was identified in a foodborne outbreak affecting 22 symptomatic high school students and 4 asymptomatic food handlers in South Korea [52].

The findings obtained in this study may have a major public health impact, despite their relatively limited clinical significance. While most cases of infection are relatively mild and self-limiting, diarrhoea due to EAEC is often persistent in children, with an average duration of about 14 days [53]. This prolonged illness can result in extended absences from school, potentially leading to significant financial strain on families, including lost income due to parents or caregivers taking time off work. Additionally, the social and behavioural impacts of prolonged school exclusion on young children should not be overlooked [54]. On the other hand, apparently healthy or asymptomatic carriers can act as unnoticed spreaders of the pathogen at the community level, putting at risk vulnerable populations including immunocompromised individuals or the elderly, in whom EAEC infection is usually more severe and may often require treatment [55, 56].

It is important to acknowledge the limitations of this study. First, as the surveyed population was geographically restricted to the Madrid area, our results and conclusions might not be representative of the epidemiological situation in other paediatric populations or regions in the country. Second, this study did not consider the testing of caregivers, food handlers, and household contacts, making impossible to fully ascertain the true sources of infection. And third, the study conducted in the infant and primary schools in the Leganés area only considered data at the school (not the classroom) level, impairing the full investigation of the EAEC transmission events.

In conclusion, data provided here suggest that carriage of certain EAEC subtypes with gastrointestinal/extra-intestinal pathogenic potential in apparently healthy Spanish children is more common than initially anticipated. In addition, these data indicate that potential transmission can occur in school settings, particularly among toddlers, although extra-school infections through alternative pathways remain possible. Further epidemiological surveys are needed to fully understand the specific role of schools in the transmission of this pathogen.

## Supplementary Information

Below is the link to the electronic supplementary material.

ESM 1 Phylogenomic hylogenomic analysis of the O3:H2-ST10 enteroaggregative Escherichia coli genomes. A SNP matrix was generated for 7 isolates with CSI Phylogeny 1.4 (https://cge.food.dtu.dk/services/CSIPhylogeny) and the published genome of E. coli strain H3 (GenBank accession no. NZ_CP028732.1) as a reference, according to KmerFinder 3.2 results. The SNP matrix was phylogenetically analyzed with RAxML 8.2.12 with a GTR model. Branch labels indicate support values for 1000 bootstrap replicates. Bootstrap values less than 90 are not shown. Monophyletic groups of isolates with a median pairwise distance of 20 or fewer SNPs, a bootstrap support of 90 or higher, and some epidemiological evidence supporting episodes of EAEC transmission are coloured in red. Unrelated study isolates are coloured in blue. Unrelated isolates from Llorente et al., available at https://doi.org/10.3389/fmicb.2023.1120285, are coloured in black. The tree scale indicates the distance of 0.001 nucleotide changes per site (PNG 15.1 KB)

High Resolution Image (TIF 65 KB)

ESM 2 Phylogenomic analysis of the O44:H18-ST1380 enteroaggregative Escherichia coli genomes. A SNP matrix was generated for 8 isolates with CSI Phylogeny 1.4 (https://cge.food.dtu.dk/services/CSIPhylogeny) and the published genome of E. coli strain SCU-105 (accession no. NZ_CP051738.1) as a reference, according to KmerFinder 3.2 results. The SNP matrix was phylogenetically analysed with RAxML 8.2.12 with a GTR model. Branch labels indicate support values for 1000 bootstrap replicates. Bootstrap values less than 90 are not shown. Monophyletic groups of isolates with a median pairwise distance of 20 or fewer SNPs, a bootstrap support of 90 or higher, and some epidemiological evidence supporting episodes of EAEC transmission are coloured in red. Unrelated isolates from Llorente et al., available at https://doi.org/10.3389/fmicb.2023.1120285, are coloured in black. The tree scale indicates the distance of 0.001 nucleotide changes per site (PNG 19.2 KB)

High Resolution Image (TIF 80 KB)

ESM 3 Phylogenomic analysis of the ONT:H33-ST34 enteroaggregative Escherichia coli genomes. A SNP matrix was generated for 7 isolates with CSI Phylogeny 1.4 (https://cge.food.dtu.dk/services/CSIPhylogeny) and the published genome of E. coli strain BR1220 (accession no. NZ_CP093068.1) as a reference, according to KmerFinder 3.2 results. The SNP matrix was phylogenetically analyzed with RAxML 8.2.12 with a GTR model. Branch labels indicate support values for 1000 bootstrap replicates. Bootstrap values less than 90 are not shown. Monophyletic groups of isolates with a median pairwise distance of 20 or fewer SNPs, a bootstrap support of 90 or higher, and some epidemiological evidence supporting episodes of EAEC transmission are coloured in red. Unrelated isolates from Llorente et al., available at https://doi.org/10.3389/fmicb.2023.1120285, are coloured in black. The tree scale indicates the distance of 0.001 nucleotide changes per site (PNG 15.4 KB)

High Resolution Image (TIF 63 KB)

Supplementary file4 Detailed description of the microbiological analysis, molecular characterization of enteroaggregative Escherichia coli isolates, and phylogenetic analysis conducted in this study (DOCX 16 KB)

Supplementary file5 (DOCX 18 KB)

Supplementary file6 (DOCX 14 KB)

Supplementary file7 Full dataset showing all the information obtained in the epidemiological questionnaires from children attending infant and primary schools in Leganés (southern metropolitan area of Madrid, central Spain) (XLSX 5616 KB)

Supplementary file8 Full dataset showing all the information obtained in the epidemiological questionnaires from children attending nursery schools in Majadahonda (northwestern metropolitan area of Madrid, central Spain) (XLSX 28 KB)

Supplementary file9 Description and genomic characteristics of the enteroaggregative Escherichia coli isolates obtained from children attending infant and primary schools in Leganés (southern metropolitan area of Madrid, central Spain) (XLSX 15666 KB)

Supplementary file10 Description and genomic characteristics of the enteroaggregative Escherichia coli isolates obtained from children attending nursery schools in Majadahonda (northwestern metropolitan area of Madrid, central Spain) (XLSX 15485 KB)

## Data Availability

FASTQ sequences were deposited in the National Center for Biotechnology Information Short Read Archive under the BioProject PRJNA913941. Accession numbers for each sequence are listed in Tables S5 and S6.

## Abbreviations

AAF: Aggregative adherence fimbriae
*aaiC*: AaiC, secreted protein
*aap*: Dispersin, antiaggregation protein
*aar*: AggR-activated regulator
*aatA*: Dispersin transporter protein
AFP: Aggregative-forming pili
AggR: Transcriptional activator
CS22: Non-fimbrial ETEC colonization factor
EAEC: Enteroaggregative *Escherichia coli*
ECDC: European Centre for Disease Prevention and Control
EEA: European Economic Area
EFSA: European Food Safety Authority
EU: European Union
ExPEC: Extraintestinal pathogenic *Escherichia coli*
MLST: Multilocus sequence typing
ONT: Non-typeable O antigen
SNP: Single nucleotide polymorphism
ST: Sequence type
UPEC: Uropathogenic *Escherichia coli*
UTI: Urinary tract infection
WGS: Whole-genome sequencing
