# Supplementary figures and images for: Molecular-based evidence for school transmission of enteroaggregative Escherichia coli among apparently healthy children attending nursery, infant, and primary schools in Madrid (Spain)
Source: Eur J Pediatr. 2025 Oct 4;184(11):658. doi: 10.1007/s00431-025-06430-z (PMC12496287; doi:10.1007/s00431-025-06430-z)

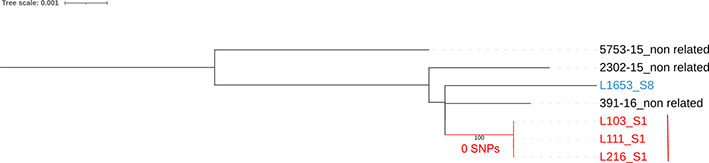

Supplement: Supplementary file 1 — Phylogenomic hylogenomic analysis of the O3:H2-ST10 enteroaggregative Escherichia coli genomes. A SNP matrix was generated for 7 isolates with CSI Phylogeny 1.4 (https://cge.food.dtu.dk/services/CSIPhylogeny) and the published genome of E. coli strain H3 (GenBank accession no. NZ_CP028732.1) as a reference, according to KmerFinder 3.2 results. The SNP matrix was phylogenetically analyzed with RAxML 8.2.12 with a GTR model. Branch labels indicate support values for 1000 bootstrap replicates. Bootstrap values less than 90 are not shown. Monophyletic groups of isolates with a median pairwise distance of 20 or fewer SNPs, a bootstrap support of 90 or higher, and some epidemiological evidence supporting episodes of EAEC transmission are coloured in red. Unrelated study isolates are coloured in blue. Unrelated isolates from Llorente et al., available at https://doi.org/10.3389/fmicb.2023.1120285, are coloured in black. The tree scale indicates the distance of 0.001 nucleotide changes per site (PNG 15.1 KB) [file 431_2025_6430_Fig4_ESM.png]

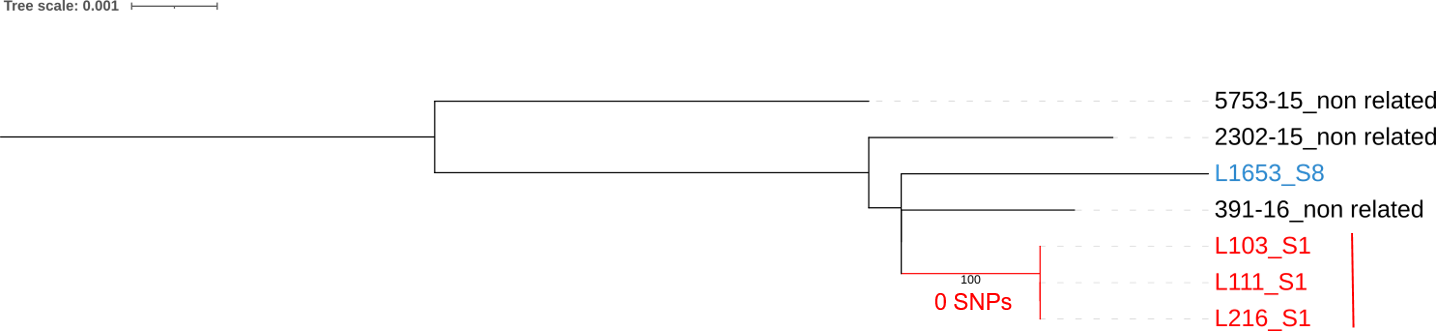

Supplement: Supplementary file 2 — High Resolution Image (TIF 65 KB) [file 431_2025_6430_MOESM1_ESM.tif]

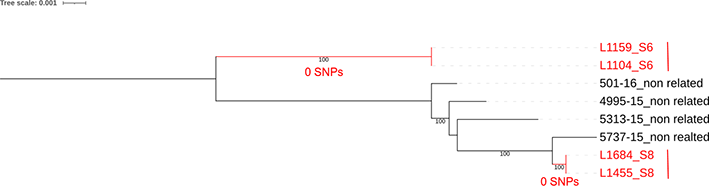

Supplement: Supplementary file 3 — Phylogenomic analysis of the O44:H18-ST1380 enteroaggregative Escherichia coli genomes. A SNP matrix was generated for 8 isolates with CSI Phylogeny 1.4 (https://cge.food.dtu.dk/services/CSIPhylogeny) and the published genome of E. coli strain SCU-105 (accession no. NZ_CP051738.1) as a reference, according to KmerFinder 3.2 results. The SNP matrix was phylogenetically analysed with RAxML 8.2.12 with a GTR model. Branch labels indicate support values for 1000 bootstrap replicates. Bootstrap values less than 90 are not shown. Monophyletic groups of isolates with a median pairwise distance of 20 or fewer SNPs, a bootstrap support of 90 or higher, and some epidemiological evidence supporting episodes of EAEC transmission are coloured in red. Unrelated isolates from Llorente et al., available at https://doi.org/10.3389/fmicb.2023.1120285, are coloured in black. The tree scale indicates the distance of 0.001 nucleotide changes per site (PNG 19.2 KB) [file 431_2025_6430_Fig5_ESM.png]

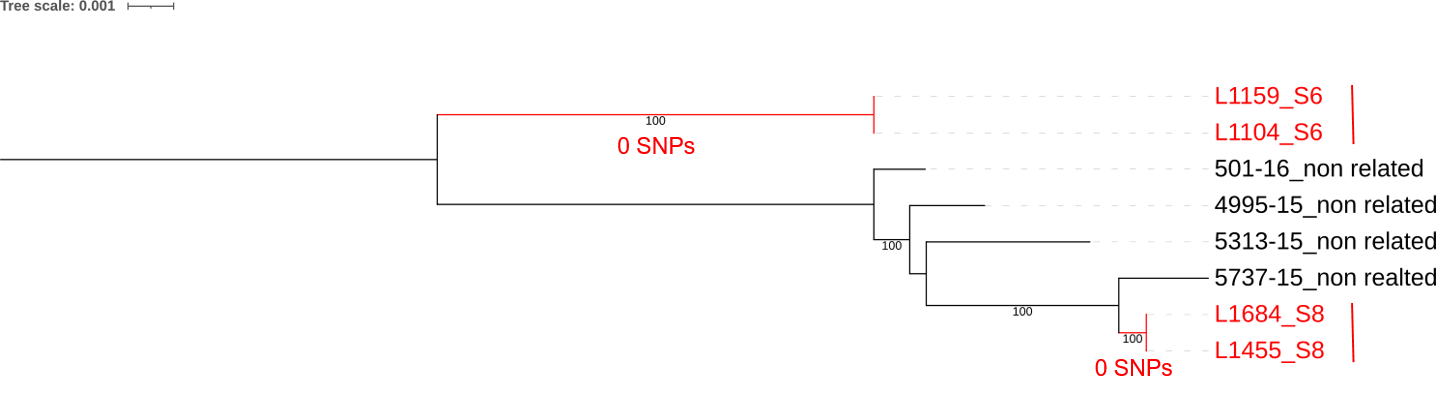

Supplement: Supplementary file 4 — High Resolution Image (TIF 80 KB) [file 431_2025_6430_MOESM2_ESM.tif]

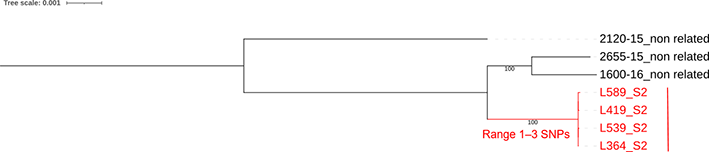

Supplement: Supplementary file 5 — Phylogenomic analysis of the ONT:H33-ST34 enteroaggregative Escherichia coli genomes. A SNP matrix was generated for 7 isolates with CSI Phylogeny 1.4 (https://cge.food.dtu.dk/services/CSIPhylogeny) and the published genome of E. coli strain BR1220 (accession no. NZ_CP093068.1) as a reference, according to KmerFinder 3.2 results. The SNP matrix was phylogenetically analyzed with RAxML 8.2.12 with a GTR model. Branch labels indicate support values for 1000 bootstrap replicates. Bootstrap values less than 90 are not shown. Monophyletic groups of isolates with a median pairwise distance of 20 or fewer SNPs, a bootstrap support of 90 or higher, and some epidemiological evidence supporting episodes of EAEC transmission are coloured in red. Unrelated isolates from Llorente et al., available at https://doi.org/10.3389/fmicb.2023.1120285, are coloured in black. The tree scale indicates the distance of 0.001 nucleotide changes per site (PNG 15.4 KB) [file 431_2025_6430_Fig6_ESM.png]

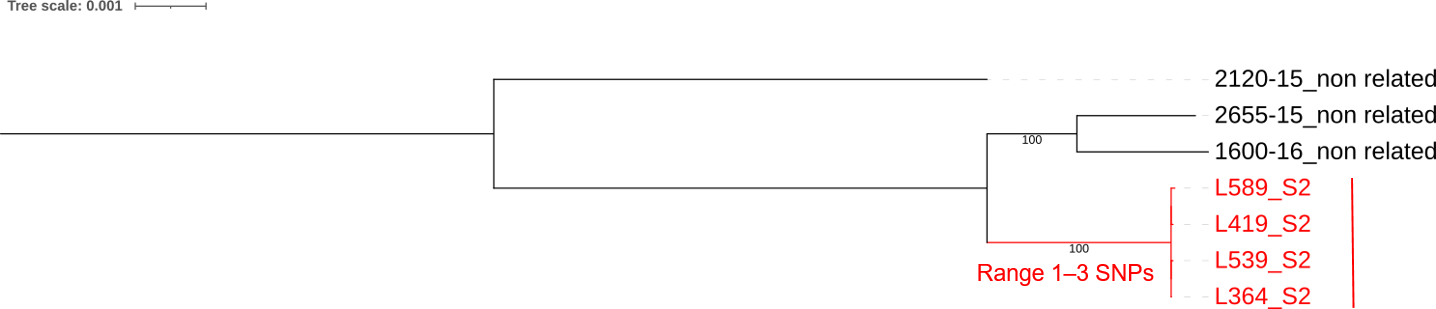

Supplement: Supplementary file 6 — High Resolution Image (TIF 63 KB) [file 431_2025_6430_MOESM3_ESM.tif]
