# Supplementary material for: Molecular-based evidence for school transmission of enteroaggregative Escherichia coli among apparently healthy children attending nursery, infant, and primary schools in Madrid (Spain)
Source: Eur J Pediatr. 2025 Oct 4;184(11):658. doi: 10.1007/s00431-025-06430-z (PMC12496287; doi:10.1007/s00431-025-06430-z)
Supplement: Supplementary file 7 — Supplementary file4 Detailed description of the microbiological analysis, molecular characterization of enteroaggregative Escherichia coli isolates, and phylogenetic analysis conducted in this study (DOCX 16 KB) [file 431_2025_6430_MOESM4_ESM.docx]

**Microbiological analysis**

Upon receipt, a stool impregnated cotton swab was inoculated in 9 ml of tryptic soy broth (TSB, Becton Dickinson, Franklin Lakes, NJ, USA) and incubated overnight at 37 °C. After this non-selective enrichment step, the TSB culture was subcultured on both MacConkey agar (MAC, Becton Dickinson) and tryptic soy agar (TSA, Becton Dickinson) and incubated overnight at 37 °C. A loopful of bacterial growth taken from the first streaking area of the TSA plate was suspended in 0.5 ml of sterile distilled water, boiled for 5 min to release the DNA, and centrifuged at 10,000 rpm for 5 min. The supernatant was used directly as a template in a conventional PCR assay using DreamTaq DNA Polymerase (Thermo Fisher Scientific, Waltham, MA, USA), and specific oligonucleotides for the amplification of a partial fragment of the *aatA* gene [1] (Table S1) in a 25 μl reaction mix. Due to the lack of a formally recognized molecular definition for EAEC and considering its historical specificity [2, 3], our diagnostic criterion for EAEC infection was the presence of the *aatA* gene. An additional *gapA*-specific PCR was also run concurrently with the diagnostic PCR assay to ensure that all samples had sufficient bacterial DNA and no PCR inhibition occurred (Table S1). Thermal cycler conditions consisted of 25 cycles of denaturation at 94 °C for 30 s, annealing at 56 °C for 40 s, and extension at 72 °C for 1 min. When culture tested EAEC-positive, up to 20 individual *E. coli*-like colonies obtained from MAC plates were tested by PCR to obtain the isolate, which was further confirmed biochemically as *E. coli* by the API 20E system (bioMérieux, Marcy l’Etoile, France).

**Whole-genome sequencing**

Genomic DNA was purified from the EAEC isolates using the NZY Tissue gDNA Isolation Kit (NZYTech, Lisbon, Portugal), according to the manufacturer’s instructions. In the case of isolates from nursery children, a DNA library was generated using the Nextera XT DNA Sample Preparation Kit (Illumina, San Diego, CA, USA) and WGS was performed with the Illumina NextSeq 500 platform (Illumina) using 300 cycles and generating 150-bp paired-end reads. As for isolates from infant and primary schoolchildren, a DNA library was generated using the Nextera DNA Flex Library Preparation Kit (Illumina) and WGS was performed with the Illumina NovaSeq 6000 platform (Illumina) also using 300 cycles and generating 150-bp paired-end reads. The reads were trimmed and filtered according to quality criteria using FastP v0.23.2 and FastQC v0.11.9, respectively [4].

**Data analysis and molecular characterization**

The O and H serogenotypes (*in silico* serotypes), virulence genes, and STs were identified by uploading the reads to SerotypeFinder v2.0, VirulenceFinder v2.0, and MLST v2.0, respectively, available on the Center for Genomic Epidemiology (CGE) website (https://cge.cbs.dtu.dk//services). The threshold of sequence identity was set to 85% and the percentage of minimum overlapping gene length to 60%. MLST tool used the seven loci (*adk*, *gyrB*, *fumC*, *icd*, *mdh*, *purA*, and *recA*) scheme. When SerotypeFinder did not predict O antigen it was considered non-typeable (ONT). The *E. coli* phylogroups were determined by uploading the assembled contigs to the ClermonTyping tool available on the Iame-research Center website (http://clermontyping.iame-research.center). The presumptive ExPEC status was assigned to those isolates positive for ≥2 of the following virulence genes: *papA* and/or *papC*, *sfa-focDE*, *afa-draBC*, *iutA*, and *kpsMII* [5]. For this purpose, isolates were considered positive for *afa-draBC* if a combination of *afaB* or *nfaE* and also *afaC* was identified by WGS and positive for *sfa-focDE* if a combination of *focC* or *sfaE* and also *focI* or *sfaD* was identified [6]. Likewise, the UPEC status was assigned to those isolates positive for ≥2 of the following genes: *chuA*, *fyuA*, *vat*, and *yfcV* [6, 7].

**Phylogenetic analysis of EAEC isolates**

To reveal possible episodes of transmission within school settings, a SNP analysis was performed for all EAEC isolates belonging to the same serogenotype-ST combinations from all settings, including unrelated isolates of the same serogenotype-ST combination obtained in a previous study [8]. The analysis was carried out by uploading the reads to CSI Phylogeny v1.4, available on the CGE website, with the following settings: a minimum depth of 10 at SNP positions, a minimum relative depth of 10% at SNP positions, a minimum distance of 10 bp between SNPs (prune), a minimum SNP quality of 30, a minimum read quality of 25, and a minimum Z-score of 1.96. According to KmerFinder v3.2 results, the published genome sequence of *E. coli* strains H3 (accession no. NZ_CP028732.1), SCU-105 (accession no. NZ_CP051738.1), A41 (accession no. NZ_CP028735.1), ESBL 15 (accession no. NZ_CP041678.1), and BR1220 (accession no. NZ_CP093068.1) were used as a reference for EAEC strains belonging to O3:H2-ST10, O44:H18-ST1380, O126:H27-ST200, O111:H21-ST40, and ONT:H33-ST34, respectively. The percentage of the reference genome covered by all isolates of the same serogenotype-ST combination ranged between 85.9% and 94.5%. From the aligned sequences of concatenated SNPs, we calculated maximum likelihood phylogenetic trees with RAxML v8.2.12 [9] with a GTR model and 1,000 bootstrap iterations. The respective consensus trees were midpoint rooted and annotated with iTOL v6 [10].

**REFERENCES**

1. Schmidt H, Knop C, Franke S, Aleksic S, Heesemann J, Karch H (1995) Development of PCR for screening of enteroaggregative *Escherichia coli*. J Clin Microbiol 33:701-705. https://doi.org/10.1128/jcm.33.3.701-705.1995

2. Beczkiewicz A, Cebelinski E, Decuir M, Lappi V, Wang X, Smith K, Boxrud D, Medus C (2019) High relative frequency of enteroaggregative *Escherichia coli* among patients with reportable enteric pathogens, Minnesota, 2016-2017. Clin Infect Dis 69:473-479. https://doi.org/10.1093/cid/ciy890

3. Pabst WL, Altwegg M, Kind C, Mirjanic S, Hardegger D, Nadal D (2003) Prevalence of enteroaggregative *Escherichia coli* among children with and without diarrhea in Switzerland. J Clin Microbiol 41:2289-2293. https://doi.org/10.1128/jcm.41.6.2289-2293.2003

4. Chen S, Zhou Y, Chen Y, Gu J (2018) fastp: an ultra-fast all-in-one FASTQ preprocessor. Bioinformatics 34:i884-i890. https://doi.org/10.1093/bioinformatics/bty560

5. Johnson JR, Murray AC, Gajewski A, Sullivan M, Snippes P, Kuskowski MA, Smith KE (2003) Isolation and molecular characterization of nalidixic acid-resistant extraintestinal pathogenic *Escherichia coli* from retail chicken products. Antimicrob Agents Chemother 47:2161-2168. https://doi.org/10.1128/AAC.47.7.2161-2168.2003

6. Malberg Tetzschner AM, Johnson JR, Johnston BD, Lund O, Scheutz F (2020) *In silico* genotyping of *Escherichia coli* isolates for extraintestinal virulence genes by use of whole-genome sequencing data. J Clin Microbiol 58:10.1128/JCM.01269-20

7. Spurbeck RR, Dinh PC, Jr., Walk ST, Stapleton AE, Hooton TM, Nolan LK, Kim KS, Johnson JR, Mobley HL (2012) *Escherichia coli* isolates that carry *vat*, *fyuA*, *chuA*, and *yfcV* efficiently colonize the urinary tract. Infect Immun 80:4115-4122. https://doi.org/10.1128/IAI.00752-12

8. Llorente MT, Escudero R, Ramiro R, Remacha MA, Martínez-Ruiz R, Galán-Sánchez F, de Frutos M, Elía M, Onrubia I, Sánchez S (2023) Enteroaggregative *Escherichia coli* as etiological agent of endemic diarrhea in Spain: A prospective multicenter prevalence study with molecular characterization of isolates. Front Microbiol 14:1120285. https://doi.org/10.3389/fmicb.2023.1120285

9. Stamatakis A (2014) RAxML version 8: a tool for phylogenetic analysis and post-analysis of large phylogenies. Bioinformatics 30:1312-1313. https://doi.org/10.1093/bioinformatics/btu033

10. Letunic I, Bork P (2019) Interactive Tree Of Life (iTOL) v4: recent updates and new developments. Nucleic Acids Res 47:W256-W259. https://doi.org/10.1093/nar/gkz239
