# Supplementary material for: Molecular-based evidence for school transmission of enteroaggregative Escherichia coli among apparently healthy children attending nursery, infant, and primary schools in Madrid (Spain)
Source: Eur J Pediatr. 2025 Oct 4;184(11):658. doi: 10.1007/s00431-025-06430-z (PMC12496287; doi:10.1007/s00431-025-06430-z)
Supplement: Supplementary file 8 — Supplementary file5 (DOCX 18 KB) [file 431_2025_6430_MOESM5_ESM.docx]

**Table S1** English version of the standardized epidemiological questionnaire used in children attending nursery schools in Majadahonda (northwestern metropolitan area of Madrid).

| **Variable** | **Category and Stata code** |
| --- | --- |
| Sex | Male (0)  Female (1) |
| Age | Integer variable (number) |
| Delivery | Vaginal delivery (0)  Caesarean section (1) |
| Feeding | Exclusively breast-fed (1)  Exclusively formula-fed (2)  Breast and formula-fed (3)  Breast and/or formula-fed and solids (4)  Only solids (weaned) (5) |
| Number of siblings | Integer variable (number) |
| Diarrhoea in the last 7 days? | No (0)  Yes (1) |
| Abdominal pain in the last 7 days? | No (0)  Yes (1) |
| Nausea in the last 7 days? | No (0)  Yes (1) |
| Vomiting in the last 7 days? | No (0)  Yes (1) |
| Reduced appetite in the last 7 days? | No (0)  Yes (1) |
| Antibiotics consumption in the last 6 months? | No (0)  Yes (1) |
| Other drugs consumption in the last 6 months? | No (0)  Yes (1) |
| Diarrhoea in family members in the last month? | No (0)  Yes (1) |
| Diarrhoea in classmates in the last month? | No (0)  Yes (1)  I don’t know (2) |
| Member of family traveling abroad in the last 6 months? | No (0)  Yes (1) |
| Member of family traveling to an EU country in the last 6 months? | No (0)  Yes (1) |
| Which countries | String variable (free text) |
| Regular contact with dogs at home? | No (0)  Yes (1) |
| Regular contact with cats at home? | No (0)  Yes (1) |
| Cats/dogs with diarrhoea at home in the last 6 months? | No (0)  Yes (1)  I don’t know (2) |
| Source of drinking water? | Tap water (0)  Bottled water (1)  Tap water and bottled water (2) |
| Practiced swimming? | No (0)  Yes (1) |
| Practiced hand washing? | Never (0)  Rarely (1)  Habitually (2)  Always (3) |
| Practiced washing of vegetables and fruits? | Never (0)  Rarely (1)  Habitually (2)  Always (3) |
