# Supplementary material for: Molecular-based evidence for school transmission of enteroaggregative Escherichia coli among apparently healthy children attending nursery, infant, and primary schools in Madrid (Spain)
Source: Eur J Pediatr. 2025 Oct 4;184(11):658. doi: 10.1007/s00431-025-06430-z (PMC12496287; doi:10.1007/s00431-025-06430-z)
Supplement: Supplementary file 9 — Supplementary file6 (DOCX 14 KB) [file 431_2025_6430_MOESM6_ESM.docx]

**Table S2** Primer pairs used for detection and isolation of enteroaggregative *Escherichia coli* in this study

| Target | Locus | Oligonucleotide^a^ | Sequence (5′–3′) | Product size (bp) | Reference |
| --- | --- | --- | --- | --- | --- |
| EAEC | *aatA* | pCDV432/start | CTGGCGAAAGACTGTATCAT | 630 | [1] |
|  |  | pCDVD432/stop | CAATGTATAGAAATCCGCTGTT |  |  |
| Control^b^ | *gapA* | gapA-F | ATCAACGGTTTTGGCCGTATC | 924 | [2] |
|  |  | gapA-R | GTTGTCGTACCAGGAYACCAG |  |  |

EAEC: enteroaggregative *E. coli*.

^a^PCR reactions contained 200 nM of each primer. Thermal cycler conditions consisted of 25 cycles of denaturation at 94°C for 30 s, annealing at 56°C for 40 s, and extension at 72°C for 1 min.

^b^Additional *gapA*-specific PCR to ensure that all samples had sufficient bacterial DNA present and no PCR inhibition occurred.

**References**

1. Schmidt H, Knop C, Franke S, Aleksic S, Heesemann J, Karch H (1995) Development of PCR for screening of enteroaggregative *Escherichia coli*. J Clin Microbiol 33(3):701-705

2. Llorente MT, Escudero R, Ramiro R, Remacha MA, Martínez-Ruiz R, Galán-Sánchez F, de Frutos M, Elía M, Onrubia I, Sánchez S (2023) Enteroaggregative *Escherichia coli* as etiological agent of endemic diarrhea in Spain: A prospective multicenter prevalence study with molecular characterization of isolates. Front Microbiol 14:1120285
